# Single‐cell combined with transcriptome sequencing to explore the molecular mechanism of cell communication in idiopathic pulmonary fibrosis

**DOI:** 10.1111/jcmm.18499

**Published:** 2024-06-18

**Authors:** Minggao Zhu, Yuhu Yi, Kui Jiang, Yongzhi Liang, Lijun Li, Feng Zhang, Xinglong Zheng, Haiyan Yin

**Affiliations:** ^1^ Intensive Care Unit The First Affiliated Hospital of Jinan University Guangzhou Guangdong China; ^2^ Department of Nephrology The First Affiliated Hospital of Jinan University Guangzhou Guangdong China

**Keywords:** biomarkers, cell communication, EMT, immune cells, IPF, single‐cell

## Abstract

Idiopathic pulmonary fibrosis (IPF) is a common, chronic, and progressive lung disease that severely impacts human health and survival. However, the intricate molecular underpinnings of IPF remains elusive. This study aims to delve into the nuanced molecular interplay of cellular interactions in IPF, thereby laying the groundwork for innovative therapeutic approaches in the clinical field of IPF. Sophisticated bioinformatics methods were employed to identify crucial biomarkers essential for the progression of IPF. The GSE122960 single‐cell dataset was obtained from the Gene Expression Omnibus (GEO) compendium, and intercellular communication potentialities were scrutinized via CellChat. The random survival forest paradigm was established using the GSE70866 dataset. Quintessential genes were selected through Kaplan–Meier (KM) curves, while immune infiltration examinations, functional enrichment critiques and nomogram paradigms were inaugurated. Analysis of intercellular communication revealed an intimate potential connections between macrophages and various cell types, pinpointing five cardinal genes influencing the trajectory and prognosis of IPF. The nomogram paradigm, sculpted from these seminal genes, exhibits superior predictive prowess. Our research meticulously identified five critical genes, confirming their intimate association with the prognosis, immune infiltration and transcriptional governance of IPF. Interestingly, we discerned these genes' engagement with the EPITHELIAL_MESENCHYMAL_TRANSITION signalling pathway, which may enhance our understanding of the molecular complexity of IPF.

## 1 INTRODUCTION

Idiopathic pulmonary fibrosis (IPF) is a chronic, progressive, fibrous interstitial lung disease^1^, ^2^ that is characterized by inflammation and scarring of pulmonary interstitial tissue,^3^ resulting in the gradual loss of pulmonary function. The exact cause is not clear, but it may be related to environmental exposure (such as silica dust, metal dust, asbestos, etc.), family genetic factors and immune abnormalities.^4^, ^5^ The prognosis of IPF is usually poor, and its survival rate mainly depends on the aetiology, severity and individual treatment response of the disease.^6^ Early diagnosis and treatment can improve the prognosis. In some cases, the condition may progress slowly, while in others, rapid progression and deterioration may occur.^7^ There are still many challenges in the treatment of IPF, and thus far, there is no complete cure for IPF.^8^ It is clear that further exploration of the molecular mechanism and treatment targets of IPF has important theoretical significance for clinical treatment.

The lung is the respiratory organs of the human body, with the basic unit responsible for gas exchange called the alveoli.^9^ Due to frequent exposure to various antigens in the air, they contain various immune cells, such as alveolar macrophages and T lymphocytes.^10^ Many studies have shown that macrophages can participate in the progression of pulmonary fibrosis through inflammatory regulation, cell clearance and repair. Liu Yuncai et al.^11^ revealed that macrophages mediate the occurrence and development of type II inflammation and fibrosis by secreting a cytokine called osteopontin, activating type II natural lymphocyte ILC2s, promoting the secretion of type II cytokines IL‐5 and IL‐13, further recruiting eosinophils and promoting goblet cells to secrete mucus. Studies have confirmed that macrophages not only play a role in the inflammatory phase but also in the receding phase of inflammation.^12^ When epithelial cells are injured, monocytes gather at the inflammatory site and differentiate into M1 macrophages under the influence of proinflammatory factors. M1 macrophages produce TNFα, IL‐1β and oxygen free radicals to kill and phagocytize microorganisms to eliminate foreign substances.^13^ These proinflammatory factors and oxygen free radicals are related to the occurrence and development of IPF.^14^ Many studies have shown that macrophages play a crucial role in the development of pulmonary fibrosis.

With the advancement of single‐cell/bioinformatics and the utilization of gene chips, microarray data analysis technology has been extensively employed in the study of various clinical diseases, aiming to identify key genes and conduct subsequent analysis.^15^, ^16^ Microarray analysis technology enables the simultaneous acquisition of expression information for tens of thousands of genes, thereby facilitating the exploration of genomic alterations associated with the occurrence and progression of diseases.^17^ Many studies have already employed bioinformatics techniques to analyse differentially expressed genes during disease progression, followed by investigations into their roles in biological processes, molecular functions and signalling pathways. These studies have contributed to the elucidation of disease pathogenesis, providing a theoretical foundation for early diagnosis and treatment.^18^, ^19^

**FIGURE 1:**
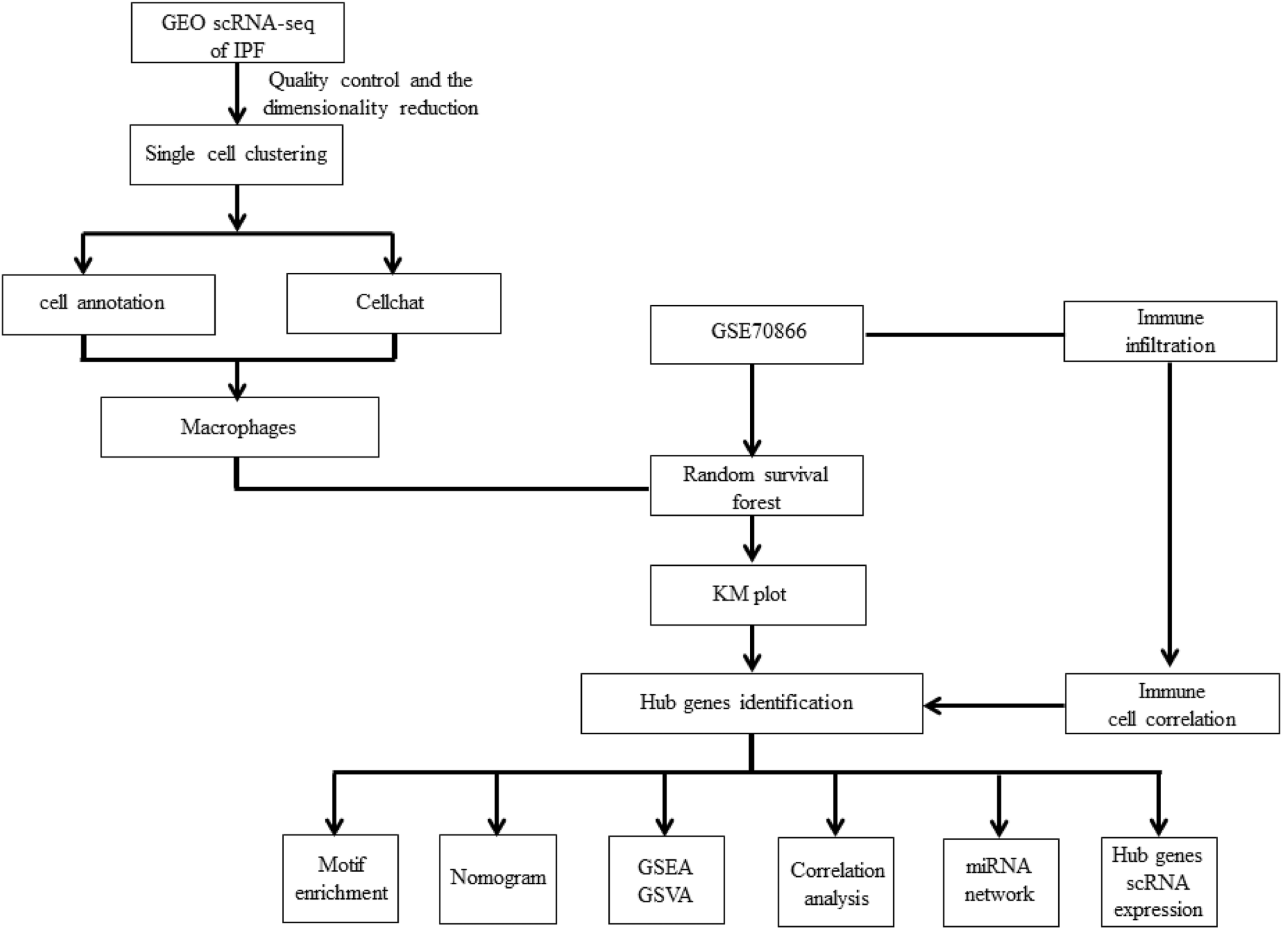
Flow chart of the analysis process conducted in this study.

In this study, single‐cell sequencing combined with transcriptome sequencing was used to compare the genetic data between patients with IPF and normal subjects. Key cell subsets were identified by the cell communication method, and important characteristic genes were screened using a random survival forest (RSF) algorithm. These findings will enhance our understanding of the underlying mechanism of IPF and provide a basis for the search for new diagnostic markers and targeted therapies. The analysis process is shown in Figure 1.

## 2 MATERIALS AND METHODS

### 2.1 Data Acquisition

The full name of the GEO database (https://www.ncbi.nlm.nih.gov/geo/info/ datasets.html) is GENE EXPRESSION OMNIBUS. This gene expression database was curated and maintained by the National Center for Biotechnology Information (NCBI) nestled within the United States' scientific edifice. For our research endeavours, we obtained the single‐cell data file GSE122960 from this lauded NCBI GEO repository, which includes expression profiles of a comprehensive set of 13 specimens. Furthermore, we retrieved the Series Matrix File data of GSE70866, accompanied by its analytical file, GPL14550. This dataset comprises expression blueprints of an aggregate of 132 patients, with 20 serving as controls and a significant 112 representing the afflicted cohort.

### 2.2 Single‐cell analysis

The expression profile was meticulously analysed via the Seurat package, filtering out genes of diminished expression (nFeature RNA > 500 & percent.mt <5). Thereafter, the dataset underwent a systematic process of standardization and harmonization and was subjected to a principal component analysis. The ElbowPlot offered a vista into the optimal number of principal components (PC), while the spatial interrelations between clusters were illuminated by UMAP scrutiny. Leveraging the celldex suite, we bestowed annotations upon a select cadre of cells pivotal to disease inception. Culminating our investigation, the hallmark genes of each cellular subtype were distilled from the single‐cell expression tableau, deploying FindAllMarkers. Those genes brandishing an absolute log2FC magnitude surpassing 1, conjoined with an adjusted *p* value plummeting beneath 0.05, were identified as the distinctive marker genes within each cellular nuance.

### 2.3 Ligand receptor interaction analysis (CellChat)

CellChat stands as an illustrious instrument, forging a path for the quantitative extrapolation and analysis of intercellular communication networks from single‐cell data. Through a blend of intricate network analysis and pattern recognition techniques, CellChat predicts the primary signalling pathways and cellular responses, elucidating the coordination of these cellular emissaries and their signal orchestrations.^20^ In this analysis, we harnessed the normalized single‐cell expression profile as the foundational input, with the cell subtype obtained from the single‐cell analysis serving as the cell information. We analysed cell‐related interactions and quantified the closeness of the interaction relationship by observing the interaction strength between cells (weights) and the number of times (count) to observe each type of cell's activity and influence in the disease.

### 2.4 Random survival forest algorithm

We utilized the RSF algorithm, a machine learning approach, to sift through hallmark genes employing the randomForestSRC suite.^21^ The prominence of prognosis‐associated genes was subsequently stratified (nrep = 1000). Genes exhibiting a relative significance exceeding 0.5 were chosen as the definitive eigengenes.

### 2.5 Immune infiltration assay

RNA‐seq data from various patient cohorts were meticulously dissected using the CIBERSORT paradigm to deduce the proportional composition of immune‐permeating cells. The ‘corrplot’ suite facilitated an examination of the symbiotic interplay among immune cells, further delving into the ramifications of their role dynamics. The ‘Vlnplot’ suite crafted depictions of the relative abundance of immune cells, appraised the genetic impact upon immune infiltration, and executed a Pearson correlation probe between gene articulation and immune cellular abundance. A threshold of *p* < 0.05 was deemed as bearing marked distinction.

### 2.6 Gene set variance analysis (GSVA)

Gene set variation analysis is an unsupervised, nonparametric strategy tailored for appraising gene set enrichment within transcriptomic tapestry. In this scholarly endeavour, gene sets were procured from the Molecular Signatures Database, with the GSVA algorithm judiciously scoring each gene ensemble, thereby elucidating the putative shifts in biological functionality across varied specimens.

### 2.7 GSEA pathway enrichment analysis

GSEA was employed to delve deeper into the disparities in signalling conduits between the cohorts of heightened and diminished expression. The foundational gene ensemble, version 7.0, sourced from the MsigDB database,^21^ served as the annotated lexicon for the subtype pathway. We embarked on an analysis contrasting the pathways across subtypes, stratifying the notably enriched gene assemblages by virtue of their congruence score (with an adjusted *p* value falling beneath 0.05).

### 2.8 MicroRNAs (miRNA) network construction

MicroRNAs stand as small noncoding RNAs orchestrating gene expression, either championing mRNA dissolution or stymying its translation. Consequently, we endeavoured to discern whether select miRNAs nested within the cardinal genes modulate the transcription or attrition of certain perilous genes. Gleaning miRNAs intricately tied to these pivotal genes from the miRcode compendium, we crafted a visual tapestry of the gene's miRNA nexus employing the finesse of Cytoscape software.

### 2.9 Transcriptional regulation analysis of key genes

In our work, we harnessed the R package ‘RcisTarget’ to prognosticate transcriptional orchestrators. Every computation orchestrated by RcisTarget emanates from the motif. We assiduously gauged the normalized enrichment score (NES) of a given motif contingent upon the aggregate motif count within the database. Beyond the annotations bequeathed by primary data, we deduced additional annotation dossiers grounded in motif congruence and genetic sequencing.

### 2.10 Nomogram model construction

We utilized a nomogram, based on regression analysis, to illustrate the complex relationship among variables within the predictive schema. Through constructing a multifaceted regression model, we assigned scores to each gradient of influencing determinants, reflecting their respective gravitas to the resultant variable (reflected in the magnitude of the regression coefficient). These scores were then coalesced to yield a cumulative tally, facilitating the computation of the anticipated value.

### 2.11 Statistical analysis

All statistical analyses were conducted using the R language (version 4.2.2), and a *p* value less than 0.05 was deemed statistically significant.

## 3 RESULTS

### 3.1 Single‐cell level analysis in scRNA‐Seq data

**FIGURE 2:**
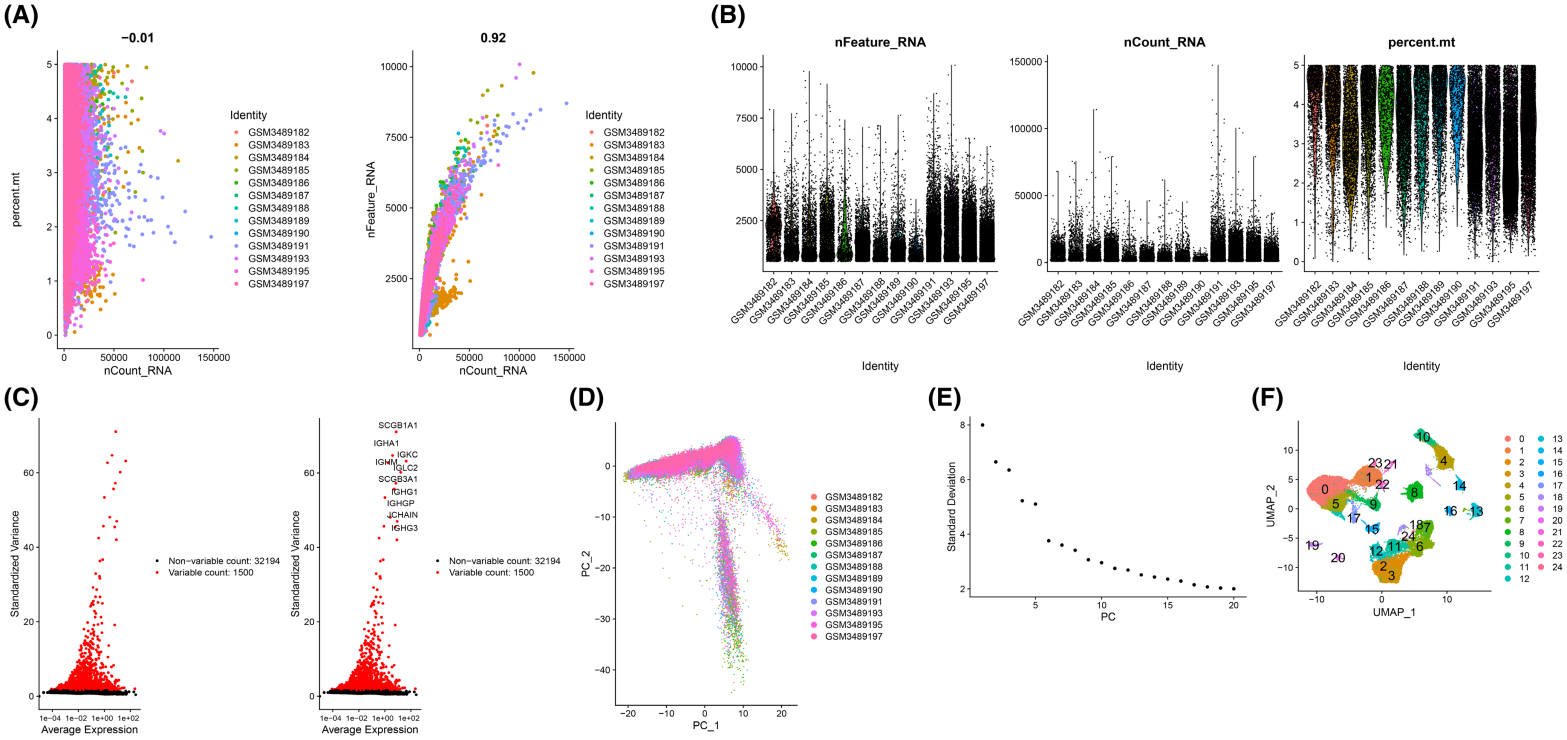
Characterization of scRNA‐Seq in idiopathic pulmonary fibrosis. (A) The left panel illustrates the relationship between cell sequencing depth and mitochondrial content, while the right panel depicts the relationship between sequencing depth and the number of genes positively correlated. Each point in the scatterplot represents an individual cell. (B) Single‐cell quality control, exhibiting the number of cells, gene count and sequencing depth per sample. Each point in the scatterplot represents an individual cell. (C) Identification of genes exhibiting significant differences between cells and visualization of characteristic variance. Each point in the scatterplot represents an individual gene. (D) Display of PCA and distribution of principal components (PC), with cells represented as dots and samples depicted by colours. Each point in the scatterplot represents an individual cell. (E) Ordering plot of variance for each PC. (F) Cell clustering into 25 clusters via the UMAP algorithm based on significant components discerned in PCA. Each colour represents a cell population identified after clustering, with the scatter points representing individual cells. The numbers in the graph indicate the cluster numbers for each group.

In this study, single‐cell data related to GSE122960 were downloaded from the NCBI GEO public database. The data samples were initially screened based on nFeature RNA and nCount RNA criteria (nFeature RNA > 500 & percent.mt <5) (Figure 2A,B), and the top 10 genes with the highest standard deviations were identified (Figure 2C). Furthermore, through PCA dimensionality reduction analysis, it was observed that the batch effect between samples was not prominent (Figure 2D), and the optimal number of PC was determined via ElbowPlot: 15 (Figure 2E). Ultimately, UMAP analysis yielded a total of 25 subgroups (Figure 2F).

### 3.2 Cell subpopulation annotation of single‐cell data

**FIGURE 3:**
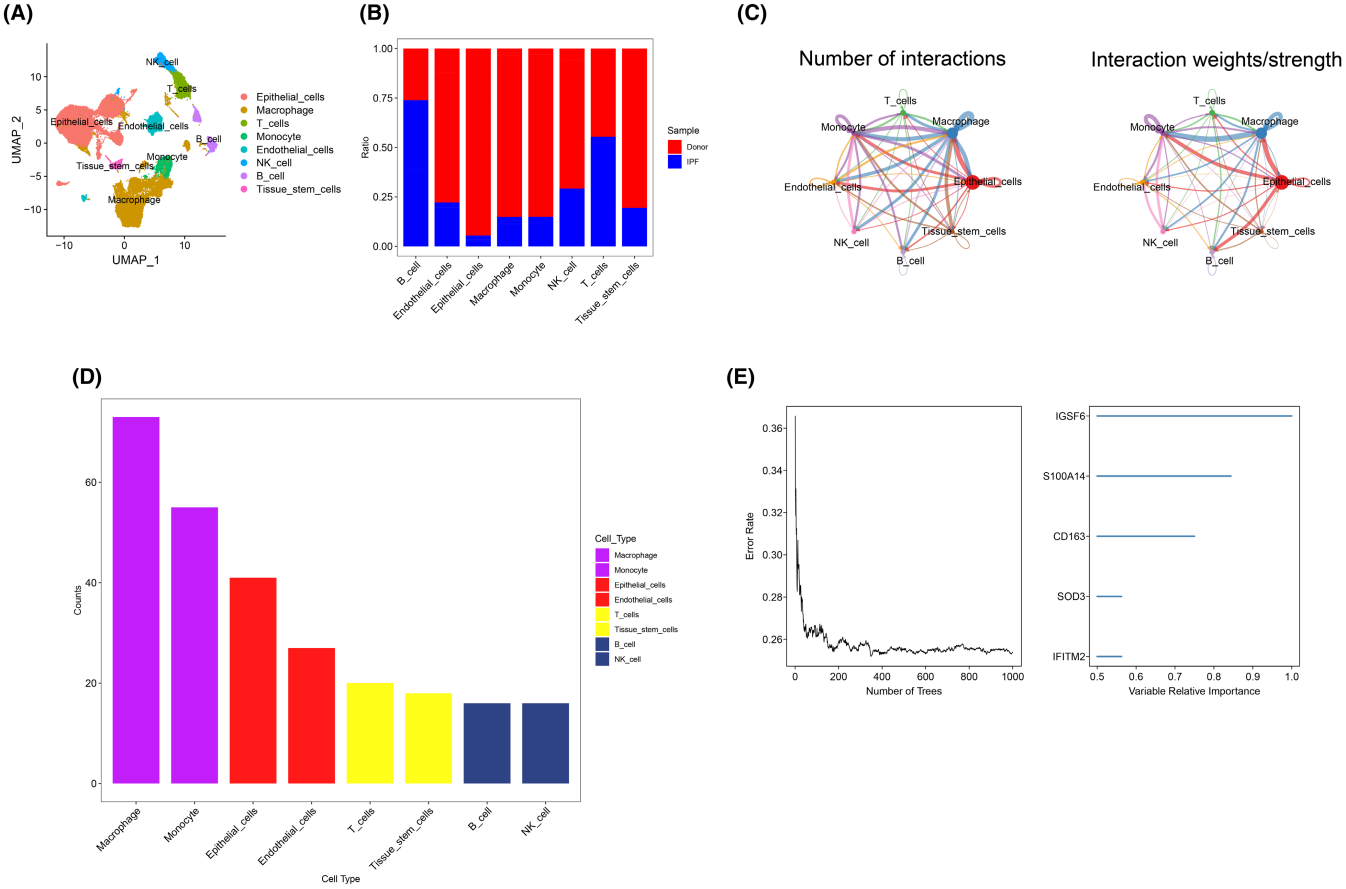
Cell Annotation and Analysis. (A) Cell annotation of 20 clusters, 20 clusters annotated into 8 cell types, epithelial cells, macrophages, T cells, monocytes, endothelial cells, NK cells, B cells and tissue stem cells. (B) The difference in the proportions of eight types of cells between healthy individuals and patients with idiopathic pulmonary fibrosis. (C) A network of cellular interactions between eight types of cells, with the edge width indicating the probability and intensity of communication between cells. (D) A comparison of the total number of interactions in the communication network between the eight cells, decreasing from left to right, with the strongest being ILC. (E) Random survival forest analysis to identify genes.

In this study, each subtype was annotated by the R package SingleR, and 25 clusters were annotated into 8 cell categories: epithelial cells, macrophages, T cells, monocytes, endothelial cells, NK cells, B cells, and tissue stem cells (Figure 3A). In addition, the differences in the content of epithelial cells, macrophages, T cells, monocytes, endothelial cells, NK cells, B cells and tissue stem cells were also explored according to the control and disease groups (Figure 3B). Finally, we extracted marker genes unique to each cell subtype (cellMarkers.txt) from the single‐cell data through the FindAllMarkers function.

### 3.3 Analysis of intercellular communication

We used the software package CellChat to analyse the ligand–receptor relationship of Fature in the single‐cell expression profile. Complex pairs of interactions were found between these cell subtypes (Figure 3C). Finally, we statistically found that cells such as macrophages and monocytes have closer potential interactions with other cells (Figure 3D). Therefore, we finally selected the marker genes of macrophages as the candidate gene set.

### 3.4 RSF model and screening of key genes

**FIGURE 4:**
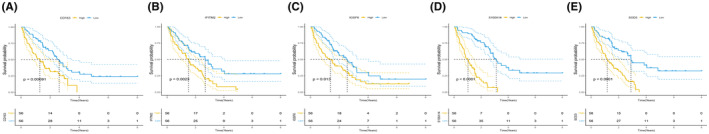
Survival Analysis of Key Genes. (A–E) Survival curves for CD163, IFITM2, IGSF6, S100A14 and SOD3 groups using the median as the cutoff point, showing statistically significant differences in survival.

To further identify the key genes affecting the progression of IPF, we performed RSF analysis on the marker genes of macrophages. We identified genes with relative importance >0.5 as final markers, showing the order of importance of the five genes (Figure 3E). Finally, we analysed the survival of these five key genes, and the results showed that CD163, IFITM2, IGSF6, S100A14 and SOD3 had statistically significant differences in survival between the high and low groups, with the median value as the cutoff point (Figure 4A–E). As a key gene for follow‐up studies.

### 3.5 Analysis of immune infiltration of key genes and their correlation with immune factors

**FIGURE 5:**
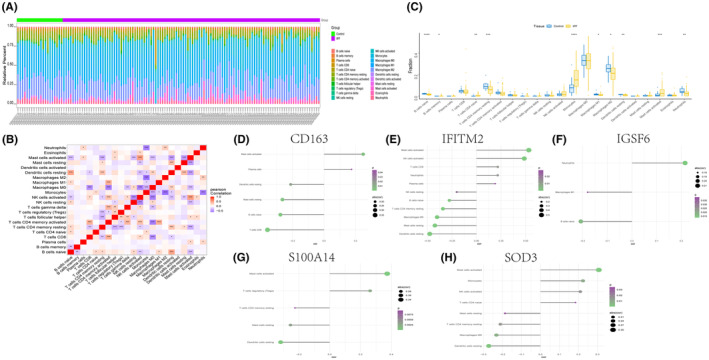
Immune infiltration in idiopathic pulmonary fibrosis (IPF) disease. (A) Relative percentages of 22 immune cell subpopulations in healthy individuals and patients with IPF. The horizontal axis represents sample names, and the vertical axis represents percentages. (B) Pearson correlations between 22 immune cells, with blue indicating negative correlation and red indicating positive correlation. One asterisk represents a *p*‐value less than 0.05, two asterisks represent a *p*‐value less than 0.01 and three asterisks represent a *p*‐value less than 0.001. (C) Differences in immune cell content between the healthy group and the IPF group. One asterisk represents a *p*‐value less than 0.05, two asterisks represent a *p*‐value less than 0.01 and three asterisks represent a *p*‐value less than 0.001. (D–H) Correlation between the expression levels of the CD163, IFITM2, IGSF6, S100A14 and SOD3 genes and immune cell content. The larger the circle, the stronger the correlations.

**FIGURE 6:**
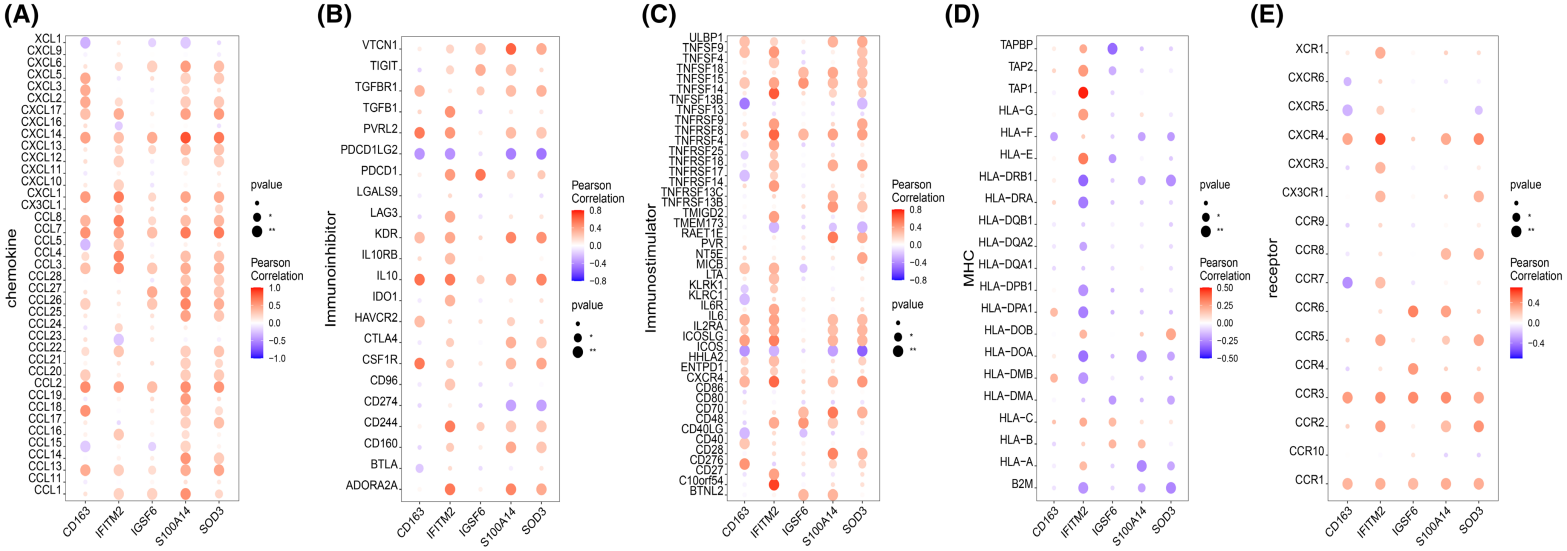
Relationship between key genes and immune factors. (A–E) Correlation of key genes with chemokines, immunoinhibitors, immunostimulators, MHC and receptors. Blue indicates negative correlation, and red indicates positive correlation. The size of the circle represents the *p*‐value, with larger circles indicating smaller *p*‐values.

The microenvironment primarily consists of fibroblasts, immune cells, the extracellular matrix, various growth factors, inflammatory factors and distinct physical and chemical characteristics. The microenvironment significantly influences disease diagnosis, survival outcomes and clinical treatment efficacy. The distribution of immune infiltration levels and the heatmap of immune cell correlation are presented in Figure 5A,B. Notably, compared to the control group, the disease group samples exhibited significantly higher levels of monocyte and mast cell activation (Figure 5C). This study further investigated the relationship between key genes and immune cells, revealing a strong correlation between several key genes and immune cells (Figure 5D–H). Additionally, the correlation between these 5 key genes and different immune factors, including immunosuppressive factors, immune stimulatory factors, chemokines and receptors, was analysed. These analyses indicated that the key genes were closely associated with the level of immune cell infiltration and played crucial roles in the microenvironment (Figure 6A–E).

### 3.6 Signalling pathways involving key genes

**FIGURE 7:**
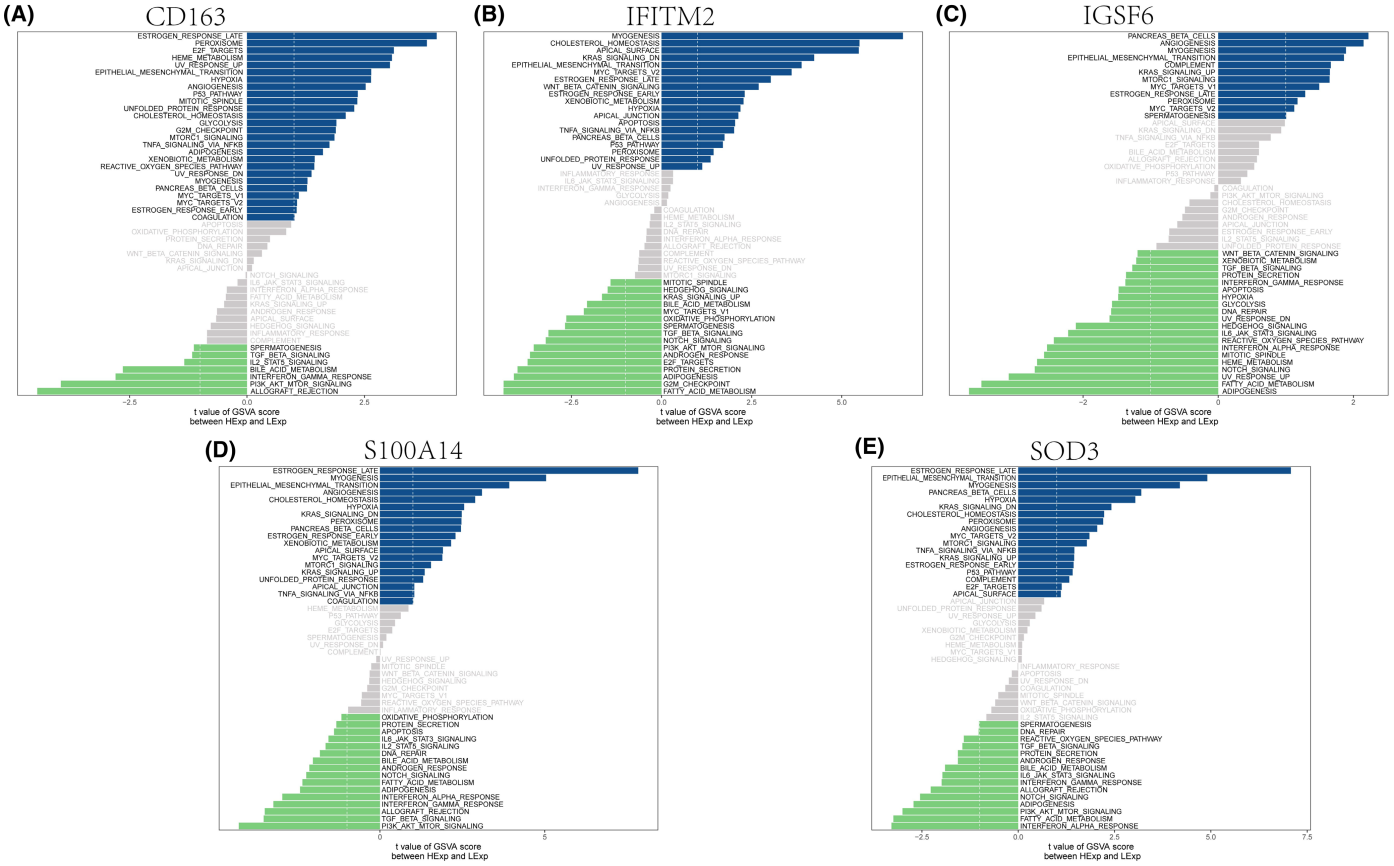
Analysis of key gene GSVA. (A–E) GSVA analysis of CD163, IFITM2, IGSF6, S100A14 and SOD3 genes showed that the signalling pathway involved in high expression of the blue genes and the signalling pathway involved in low expression of the green genes. The background gene set was a hallmark.

**FIGURE 8:**
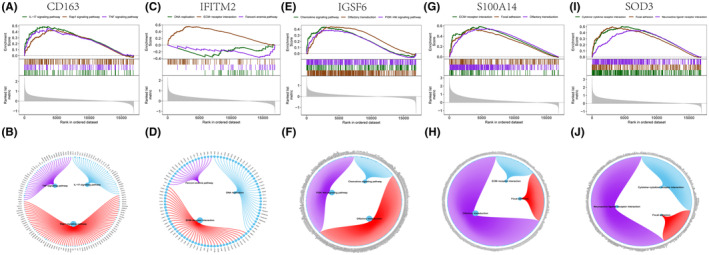
GSEA of key genes. (A–J) KEGG signalling pathways involved in the CD163, IFITM2, IGSF6, S100A14 and SOD3 genes, as well as pathway regulation and involved genes. The y‐axis represents the Enrichment Score, while the x‐axis represents each gene within this gene set, with each vertical line corresponding to each gene analysed.

Next, we will study the specific signalling pathways involving the five key genes and explore the underlying molecular mechanism by which the key genes affect the progression of IPF. GSVA results showed that high expression of CD163 can be enriched in signalling pathways such as EPITHELIAL_MESENCHYMAL_ TRANSITION, OESTROGEN_RESPONSE_LATE, P53_PATHWAY and REACTIVE_ OXYGEN_SPECIES_PATHWAY (Figure 7A). High expression of IFITM2 can be enriched in signalling pathways such as CHOLESTEROL_HOMEOSTASIS, OESTROGEN_RESPONSE_LATE, EPITHELIAL_MESENCHYMAL_TRANSITION and WNT_BETA_CATENIN_SIGNALLING (Figure 7B). High expression of IGSF6 was enriched in EPITHELIAL_MESENCHYMAL_TRANSITION, KRAS_SIGNAL ING_UP, MTORC1_SIGNALLING and OESTROGEN_RESPONSE_LATE (Figure 7C). High expression of S100A14 was enriched in EPITHELIAL_MESENCHYMAL_ TRANSITION, OESTROGEN_RESPONSE_LATE, ANGIOGENESIS and KRAS_ SIGNALLING_UP (Figure 7D). High expression of SOD3 was enriched in OESTROGEN_RESPONSE_LATE, EPITHELIAL_MESENCHYMAL_TRANSITION, KRAS_SIGNALLING_UP, MYC_TARGETS_V2 and OESTROGEN_RESPONSE_ EARLY (Figure 7E). In addition, GSEA showed that CD163 was enriched in the IL − 17 signalling pathway, Rap1 signalling pathway, and TNF signalling pathway (Figure 8A, B); IFITM2 was enriched in DNA replication, ECM − receptor interaction, and Fanconi anaemia pathway (Figure 8C,D); IGSF6 was enriched in the chemokine signalling pathway, olfactory transduction and PI3K − Akt signalling pathway (Figure 8E,F); S100A14 was enriched in the ECM − receptor interaction, focal adhesion, olfactory transduction and other pathways (Figure 8G,H); and SOD3 was enriched in pathways such as cytokine−cytokine receptor interaction, focal adhesion and neuroactive ligand−receptor interaction (Figure 8I,J). This suggests that key genes may affect gene progression through these pathways.

### 3.7 Construction of the miRNA network and analysis of transcriptional regulation related to key genes

**FIGURE 9:**
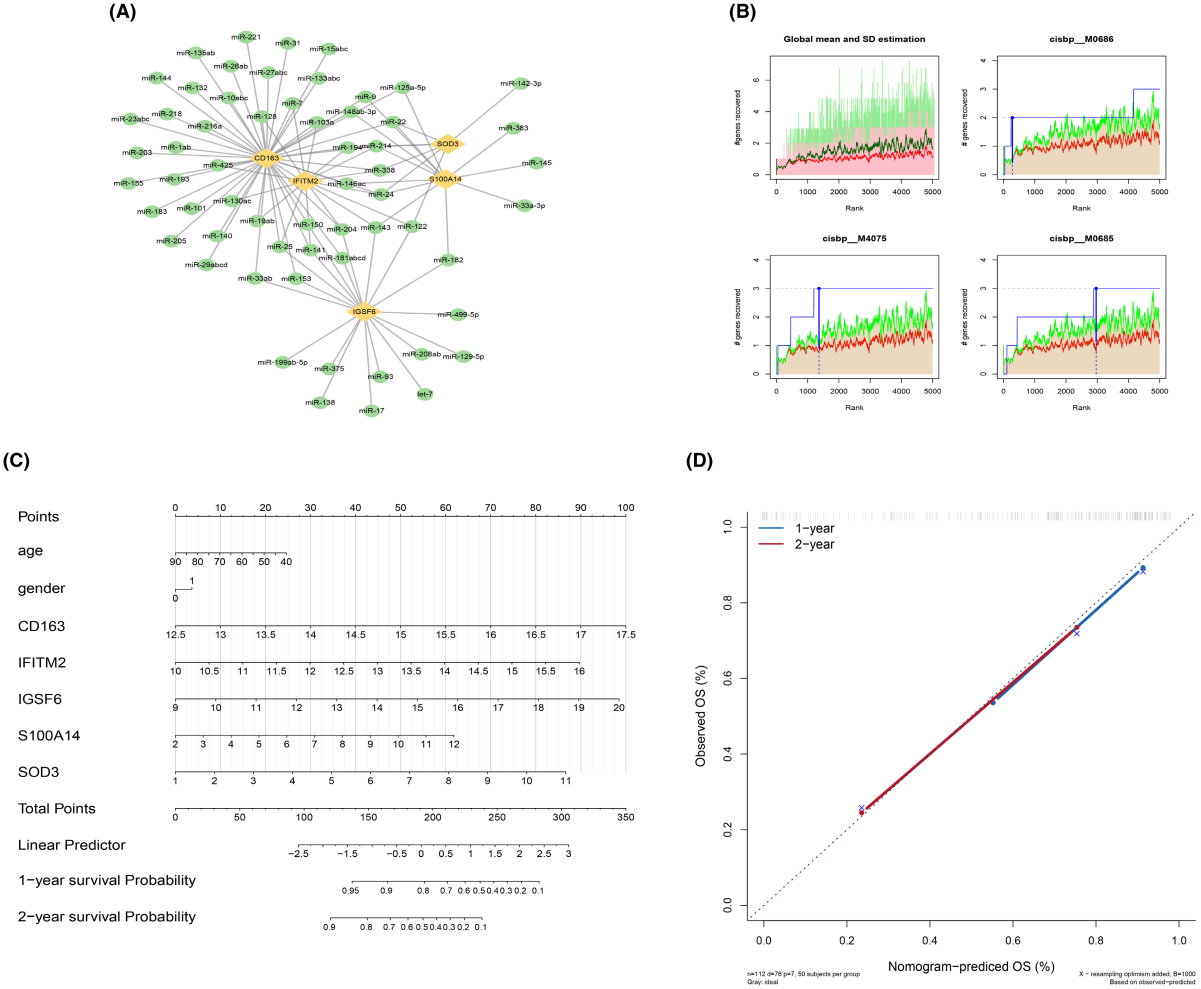
Motif transcriptional regulation analysis of core genes and nomogram model construction. (A) miRNA networks of key genes, with yellow representing mRNA and green representing miRNA. (B) The four motifs with higher AUC. The red line in the figure is the mean value of the recovery curve of each motif, the green line is the mean value + standard deviation, and the blue line is the recovery curve of the current motif. The maximum distance between the current motif and the green curve (mean + sd). (C) The values of different clinical indicators of idiopathic pulmonary fibrosis and the expression distribution of CD163, IFITM2, IGSF6, S100A14 and SOD3 contributed to different degrees in the whole scoring process. (D) Forecast analysis of the OS situation for one‐ and two‐year periods.

Through screening with the miRcode database, 5 key genes were identified and subjected to reverse prediction, resulting in the identification of 60 miRNAs and a total of 97 mRNA–miRNA relationship pairs. These relationships were visualized using Cytoscape (Figure 9A). The gene set analysed in this study focused on these 5 key genes, revealing common regulatory mechanisms, such as multiple transcription factors. Enrichment analysis of these transcription factors was performed using recovery curves, MotifF annotation and selection analysis of important genes. The analysis results indicated that the cisbp__M0686 motif had the highest normalized enrichment score (NES: 6.58). An overview of all enriched motifs and their corresponding transcription factors for the key genes is displayed in Figure 9B.

### 3.8 Construction of the nomogram model

We present the results of regression analysis in the form of a nomogram through the expression levels and clinical information of CD163, IFITM2, IGSF6, S100A14 and SOD3. The results of regression analysis show that in all our samples, IPF the values of different clinical indicators and the expression distribution of CD163, IFITM2, IGSF6, S100A14 and SOD3 had different degrees of contribution in the whole scoring process (Figure 9C). At the same time, we also carried out predictive analysis on OS in two periods of 1 year and 2 years (Figure 9D). The results showed that the nomogram models related to CD163, IFITM2, IGSF6, S100A14 and SOD3 had better predictive performance.

### 3.9 Correlation between key genes and disease regulatory genes

**FIGURE 10:**
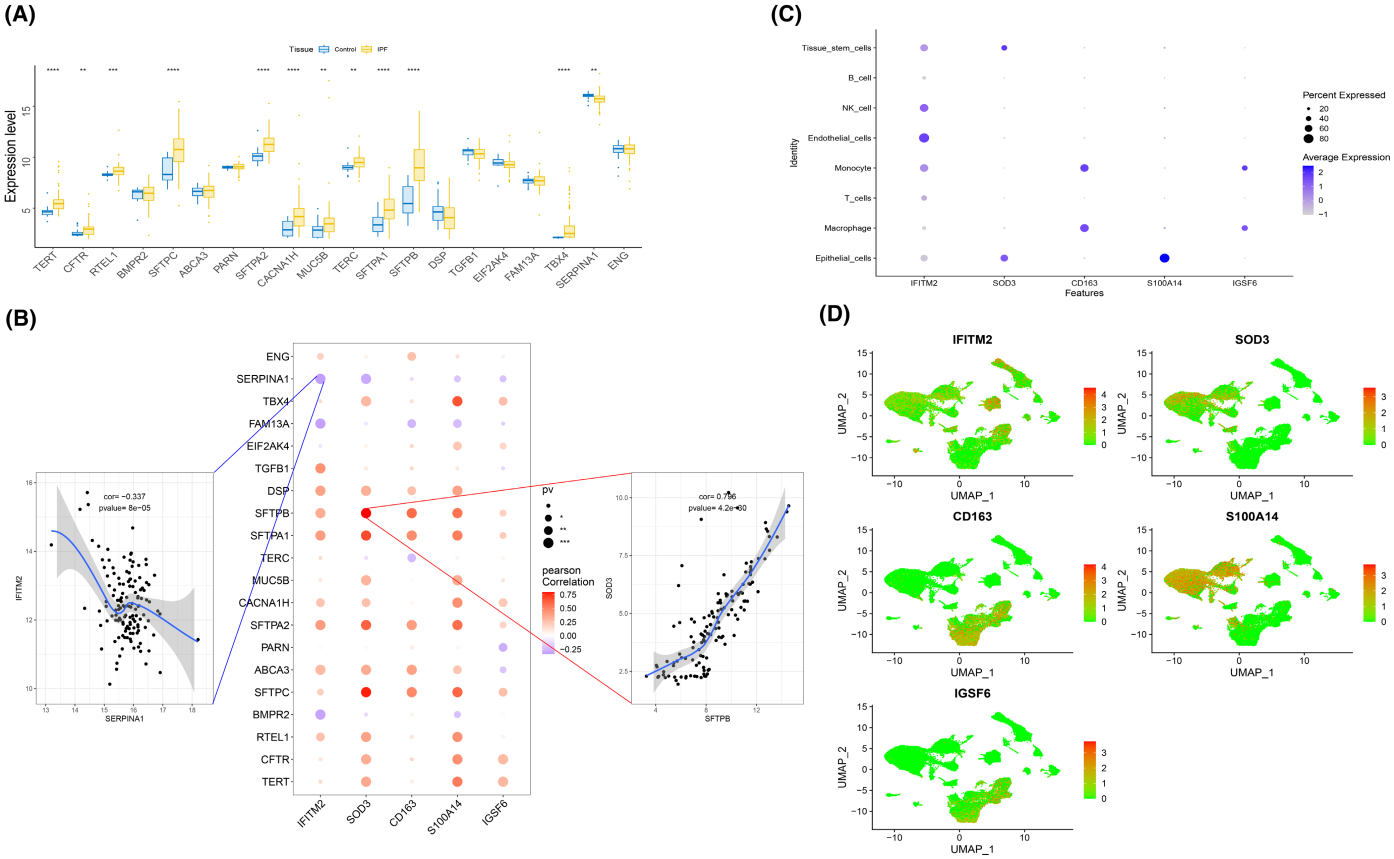
Overview of key gene expression in single cells. (A) Differences in the expression of disease‐regulating genes in idiopathic pulmonary fibrosis (IPF), with blue representing healthy individuals group and yellow representing IPF group. One asterisk represents a *p*‐value less than 0.05, two asterisks represent a *p*‐value less than 0.01, and three asterisks represent a *p*‐value less than 0.001. (B) Spearman correlation analysis of key genes and disease genes, with blue indicating a negative correlation and red indicating a positive correlation. Blue indicates negative correlation, while red indicates positive correlation. The larger the circle, the smaller the *p*‐value. (C–D) Overview of key gene expression in cells. Blue represents high expression, and grey represents low expression. The size of the circle represents the percentage it occupies.

In this study, the genes related to the disease‐regulated genes of IPF were obtained through the GeneCards database (https://www.genecards.org/). The expression levels of the top 20 genes according to the relevance score were analysed, and it was found that the expression levels of TERT, CFTR, RTEL1, SFTPC, SFTPA2, CACNA1H, MUC5B, TERC, SFTPA1, SFTPB, TBX4 and SERPINA1 were different between the two groups of patients (Figure 10A). In addition, the expression levels of five key genes were found to be significantly correlated with those of disease‐regulated genes (Figure 10B), among which SOD3 and SFTPB were significantly positively correlated (*r* = 0.796), and IFITM2 and SERPINA1 were significantly negatively correlated (*r* = −0.337).

### 3.10 Expression status and co‐expression analysis of key genes in single cells

**FIGURE 11:**
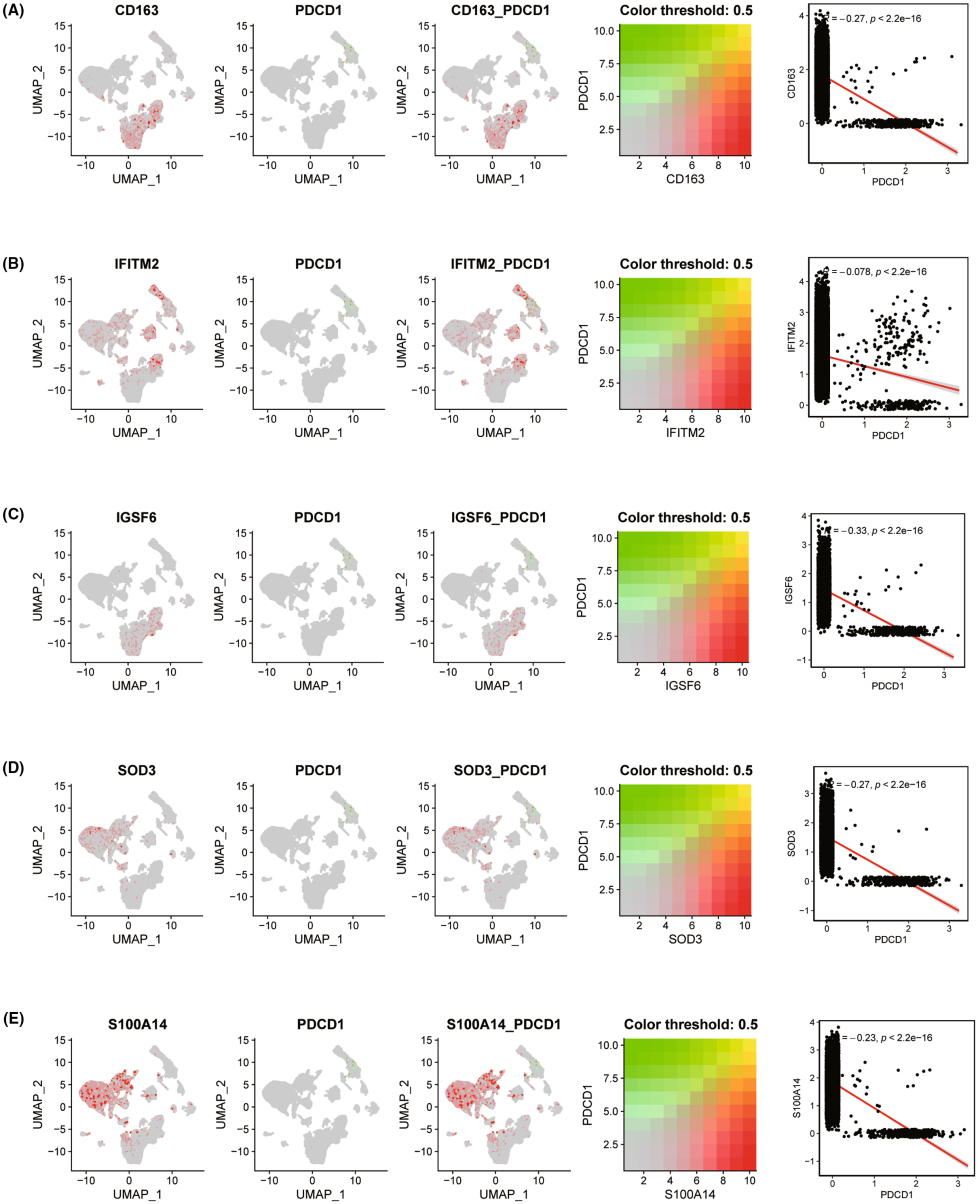
Co‐expression analysis of key genes in single cells. (A–E) Gene co‐expression of PD‐1 and key genes in single‐cell data and co‐expression gene correlation.

In this study, the expression of key genes was analysed in single cells. The expression levels of CD163, IFITM2, IGSF6, S100A14 and SOD3 in epithelial cells, macrophages, T cells, monocytes, endothelial cells, NK cells, B cells and tissue stem cells are shown in Figure 10C,D. In addition, the gene co‐expression of PD‐1 and key genes in the single‐cell data and the correlation of co‐expressed genes are also shown (Figure 11A–E).

## 4 DISCUSSION

Idiopathic pulmonary fibrosis is the most common and common type of pulmonary fibrosis.^22^ Global statistics indicate that five million individuals suffering from IPF worldwide.^23^ With exacerbating factors like environmental pollution and novel coronavirus infection,^24^ the incidence rate is on the rise.^25^ Despite considerable research efforts dedicated to IPF, treatment options remain severely limited, with no medications demonstrating significant efficacy in extending patient survival.^26^ The lack of therapeutic drugs is mainly due to the unclear pathogenesis of IPF.^27^ Hence, it is still critical and urgent to screen new biological targets to help develop patient‐specific treatments and improve prognosis.

Initially, we identified eight types of cells in the scRNA‐seq spectrum from patients with pulmonary fibrosis patients: epithelial cells, macrophages, T cells, monocytes, endothelial cells, NK cells, B cells and tissue stem cells. Notably, these cells exhibit frequent intercellular communication. We observed that cells such as macrophages and monocytes have closer potential interactions with other cells. Consequently, we finally chose the macrophage marker gene as the candidate gene set. We analysed the marker genes of macrophages by RSF analysis and identified the five most related key genes (CD163, IFITM2, IGSF6, S100A14 and SOD3). Subsequent Kaplan–Meier analysis revealed a significant correlation between these five key genes and IPF prognosis. Furthermore, we constructed the nomogram model, and the calibration curve verified the good predictive effect of five key genes, which emerged as independent factor affecting the prognosis of IPF.

Immune cell infiltration serves as a pivotal pathogenic determinant in respiratory diseases.^28^, ^29^, ^30^ Increasing evidence has demonstrated that the pattern of immune cell infiltration is closely related to pulmonary fibrosis.^31^, ^32^ Notably, our study corroborates the significant involvement of macrophages in the pulmonary fibrosis process.^33^, ^34^ Macrophages increase the clearance rate of pathogens and play a key role in the immune response to antifibrosis.^35^ Consequently, we analysed the correlation between five key genes and immune cells and found that several key genes were highly correlated with immune cells. Moreover, our analysis extended to exploring the correlation between five key genes and different immune factors, such as immunosuppressive factors, immunostimulatory factors, chemokines and receptors. These comprehensive analyses show that key genes not only have predictive value for IPF but are also closely related to the permeability of immune cells and play an important role in the immune microenvironment.

Some of the five key genes have demonstrated associations with lung diseases. For instance, CD163 has been confirmed to be overexpressed on alveolar macrophages in patients with severe chronic obstructive pulmonary disease.^36^ Yamashita et al.^37^ reported that the CD163 expression profile can be used as a biomarker to distinguish IPF from idiopathic nonspecific interstitial pneumonia. Overexpression of S100A14 can lead to malignant progression and predict poor prognosis of lung adenocarcinoma.^38^ The data suggest that SOD3 and extracellular oxidative stress may play an important role in the development of pneumoconiosis and pulmonary vascular remodelling after exposure to environmental and occupational silica.^39^ Interestingly, our study found that the epithelial‐mesenchymal cell transformation (EMT) signalling pathway is activated when five key genes are highly expressed. EMT is a biological process in which differentiated alveolar epithelial cells lose their epithelial characteristics and acquire mesenchymal cell morphology and migration. It has been established that EMT is a key step in the pathogenesis of pulmonary fibrosis.^40^, ^41^ Therefore, we speculate that these key genes may promote the occurrence and development of IPF by activating the epithelial‐mesenchymal transformation signalling pathway. In addition, oestrogen response late signalling pathways are activated when CD163, S100A14 and SOD3 are overexpressed. Studies have confirmed that oestrogen, as a self‐antioxidant, can inhibit the occurrence and development of liver fibrosis.^42^ Therefore, we speculate that CD163, S100A14 and SOD3 may affect the occurrence and development of IPF by activating the oestrogen response late signalling pathway. Pulmonary surfactant protein A1 (SFTPA1), a member of the type C lectin subfamily, is mainly synthesized in type II alveolar cells of the lung. Its function is to reduce the surface tension of alveoli, prevent alveoli from collapsing during exhalation, and play a key role in maintaining lung homeostasis and the innate immune response. Interruption of SFTPA1 can lead to a variety of acute or chronic lung diseases.^43^ For example, Takezaki et al.^44^ found that a homozygous SFTPA1 mutation drives type II lung and alveolar epithelial cell necrotic apoptosis in patients with IPF, and SFTPA1 is used as a biomarker of anti‐fibrosis drug treatment in patients with IPF. Lin et al.^45^ confirmed that the pulmonary surfactant protein genes SFTPA1 and SFTPB are genetically associated with cystic fibrosis. Desroziers et al.^46^ reported that the subform pathogenic variation in SFTPB leads to adult pulmonary fibrosis. We found that SOD3 has a strong positive correlation with SFTPB and SFTPA1, so we speculate that SOD3 may interact with FTPB and SFTPA1 to affect the occurrence and development of IPF. Tang et al.^47^ reported the potential application of immune checkpoints, especially the PD‐1/PD‐L1 pathway, in the treatment of IPF, suggesting the possibility of utilizing these pathways as new therapeutic directions. Celada et al.^48^ reveals upregulation of PD‐1 expression in IPF patients and indicates that PD‐1 blockade can reduce the expression of fibrosis‐related cytokines and collagen production, thereby potentially playing a role in therapy. PD1 may not only play a crucial role in tumour immunotherapy but also could be a key factor in regulating immune responses and the fibrotic process in IPF. By investigating the relationship between target genes and PD1 expression, our study provides a new perspective on the immune infiltration characteristics of IPF and offers scientific evidence for the development of new treatment strategies. This further underscores the potential importance of immune checkpoint inhibitors in treating IPF and opens up new directions for future research. In conclusion, our research reveals that macrophages play a crucial role in the development of IPF and screens out five key genes. Then, we further explored the immune infiltration, transcriptional regulation and signalling pathways of the key genes. This provides a new perspective for molecular mechanism research on IPF.

## 5 CONCLUSION

The heterogeneity of IPF, the interaction of each cell population and immune osmosis are considered to the maximum. The significance of this study lies in the identification of five key genes that exhibit enhanced predictive capability for IPF prognosis. Additionally, and further analyse the specific signalling pathways involved in these key genes and their relationship with pulmonary fibrosis regulatory genes. Overall, this study offers fresh information and insights for the investigation of genes linked to IPF. However, this study has several inevitable limitations: (1) the sample size of scRNA‐seq data is relatively small; (2) the regulatory mechanism of target genes in pulmonary fibrosis necessitate further exploration and validation through basic experiments, which is the future work proposed in this study that should continue to explore.

## AUTHOR CONTRIBUTIONS

**Minggao Zhu:** Conceptualization (lead); formal analysis (lead); writing – original draft (equal); writing – review and editing (equal). **Yuhu Yi:** Resources (equal); software (equal); supervision (equal); validation (equal); writing – original draft (equal). **Kui Jiang:** Data curation (equal); formal analysis (equal); methodology (equal); writing – original draft (equal). **Yongzhi Liang:** Resources (equal); software (equal); writing – original draft (equal). **Lijun Li:** Software (equal); visualization (equal); writing – original draft (equal). **Feng Zhang:** Funding acquisition (equal); project administration (equal); validation (equal). **Xinglong Zheng:** Conceptualization (equal); data curation (equal); formal analysis (equal); investigation (equal). **Haiyan Yin:** Conceptualization (lead); data curation (lead); visualization (equal); writing – review and editing (equal).

## FUNDING INFORMATION

This work was sponsored by the Medical Scientific Research Foundation of Guangdong Province, China (Number: A2021176).

## CONFLICT OF INTEREST STATEMENT

The authors declare that the research was conducted in the absence of any commercial or financial relationships that could be construed as a potential conflict of interest.

## CONSENT FOR PUBLICATION

All authors have made a substantial contribution to this article and consent to publication.

## Data Availability

The datasets presented in this study can be found in online repositories. The names of the repositories/repositories and accession number(s) can be found in the article/supplementary material.
